# Functional ultrasound assessment of cerebral blood flow and brain connectivity in a pilocarpine-induced acute epileptic seizures in mice

**DOI:** 10.3389/fneur.2026.1739882

**Published:** 2026-06-15

**Authors:** Yao Liu, Chang Xu, Wenqian Ma, Dan Zhao, Baocong Yu, Rui Zhang, Haiyue Huang, Jianguo Niu, Ling He, Yujun Wen

**Affiliations:** 1 Ningxia Key Laboratory of Craniocerebral Diseases, Ningxia Medical University, Yinchuan, China; 2 Department of Anesthesiology and Perioperative Medicine, First School of Clinical Medicine, Ningxia Medical University, Yinchuan, China; 3 Department of Human Anatomy, School of Basic Medical Sciences, Ningxia Medical University, Yinchuan, China; 4 Department of Obstetrics, Peking University First Hospital-Ningxia Women and Children’s Hospital, Teaching Hospital of Ningxia Medical University, Yinchuan, China

**Keywords:** acute epileptic seizures, cerebral hemodynamics, functional ultrasound imaging, neurovascular response, resting-state functional connectivity

## Abstract

**Background:**

Temporal lobe epilepsy (TLE) is one of the most prevalent pharmaco-resistant epilepsies in adults, for which no effective curative regimens or disease-modifying therapies are currently available. Studies have shown that cerebral blood volume (CBV) is significantly reduced throughout the whole brain in epileptic patients during the interictal period, while some have reported increased local cerebral blood flow (CBF). These findings indicate that during epileptic seizures, there are changes in the blood flow of the brain. However, for TLE, the changes in brain blood flow and the functional connections of related nuclei remain unclear.

**Methods:**

The present study aimed to use functional ultrasound imaging (fUS) to detect changes in CBV in mice during acute seizure state of TLE, as well as alterations in global brain functional network connectivity.

**Results:**

Our findings showed that most brain regions exhibit changes in relative CBV (rCBV). Among the 12 epilepsy-related brain regions, all except the intermediodorsal nucleus of the thalamus (IMD) and striatum (STR) showed an initial increase followed by a decrease in rCBV. Additionally, the rCBV changes in the reticular nucleus of the thalamus (RT) occurred earlier than in other nuclei, the brain’s functional connectivity (FC) underwent distinct changes, which may be associated with the remodeling of brain FC.

**Conclusion:**

These results demonstrated that CBV exhibited heterogeneous changes across the whole brain, and the overall brain functional network connectivity underwent modifications during acute seizure state of TLE in mice.

## 1 Introduction

Epilepsy is a common chronic central nervous system disorder characterized by abnormal synchronous neuronal activation. Repeated epileptic seizures, together with the associated injuries and complications, impose a substantial burden on patients and society (1). Recent studies have emphasized the critical role of the vascular system in temporal lobe epilepsy (TLE) (2). Researchers used functional magnetic resonance imaging (fMRI) to assess cerebral surface perfusion and observed a significant reduction in global cerebral blood flow (CBF) in patients with TLE, indicating sustained long-term hypoperfusion. This finding suggests that epilepsy may induce structural or functional changes in cerebral blood vessels (3). In another study, transcranial Doppler ultrasonography (TCD), a technique used to estimate CBF by measuring arterial blood flow velocity, revealed a significant increase in TCD speed during epileptic seizures. This elevation facilitates the detection and management of status epilepticus (4). Notably, cerebrovascular dysfunction can both trigger and sustain seizures, and restoring cerebral blood volume (CBV) integrity is considered a key mechanism of action for antiepileptic drugs (5). Collectively, these findings highlight the important role of cerebral hemodynamics in epileptic seizures.

Functional ultrasound imaging (fUS) offers superior spatiotemporal resolution compared to fMRI and positron emission tomography (PET). It also outperforms optical microscopy techniques in deep-brain imaging (6, 7). A key advantage of fUS is its ability to visualize transient whole-brain blood volume changes, making it well-suited for studying complex, short-lived events such as epileptic seizures (8). Using high-speed plane wave excitation, fUS enables high-resolution, real-time imaging of dynamic microvascular responses across the entire brain during neural activation (9). In recent years, fUS combined with other techniques has been widely applied in basic and clinical research. For example, fUS integrated with deep brain stimulation (DBS) has been used to monitor rodent responses to neural stimulation (10). Other applications include developing technologies that link ultrasound to molecular-level neural activity (11), spinal cord imaging (12), neural circuits studies (13), and cerebrovascular diseases research (14), among others.

The present study aimed to use fUS to detect changes in CBV in mice, as well as alterations in global brain functional network connectivity. We found that during acute seizure state of TLE in mice, CBV exhibited heterogeneous changes across the whole brain, the overall brain functional network connectivity underwent modifications.

## 2 Materials and methods

### 2.1 fUS

fUS data were acquired using the Iconeus One system (Iconeus, Paris, France), which is specifically designed for animal studies (15). Ultrasound images were acquired through the mice’s heads using a 15 MHz ultrasound probe connected to an ultrafast ultrasound scanner. The probe was mounted on a linear motor stage (SLC-1740, SmarAct GmbH) with a travel range of 26 mm, enabling precise positioning and scanning of the brain. Target plane images were obtained by selecting the 62nd, 66nd, and 70nd images from the Allen Brain Atlas. Mice were securely fixed on an iron frame, and the ultrasonic probe was coupled to the mice’s heads using acoustic coupling gel to complete the imaging setup. fUS data acquisition involved emitting 11 tilted plane waves (ranging from −10° to +10°) at a pulse repetition frequency of 5.5 kHz. The imaging sequence and real-time Doppler reconstruction were performed using dedicated acquisition software (IcoScan). For 3D fUS imaging, data were collected at three distinct positions with a 0.6 s temporal interval and 0.4 mm spatial spacing between positions. This resulted in a final volumetric frame rate of 0.4 Hz, with each full volume captured over 1.8 s.

### 2.2 Animals

Fifty male C57BL/6J mice (6–8 weeks old, weighing approximately 20 g during the study) were used in the experiment. The mice were housed under controlled environmental conditions (temperature: 22 ± 2 °C; relative humidity: 50%; light/dark cycle: 12 h/12 h) with ad libitum access to food and water. To minimize stress during the experiment, the mice were allowed a 7-day adaptation period after surgery. The animal groups are as follows: EEG control group of 5 mice, TLE group of 5 mice, behavioral testing control group of 10 mice, TLE group of 10 mice, c-Fos immunofluorescence staining control group of 5 mice, TLE group of 5 mice, fUS testing of a total of 20 mice. During the experiment, mice that died or showed significantly abnormal experimental results were excluded from the final data analysis to ensure the reliability and validity of the research findings.

All animal experiments were performed in accordance with the ethical guidelines for animal experimentation of Ningxia Medical University. The animal study protocol was approved by the Institutional Animal Care and Use Committee (IACUC) of Ningxia Medical University (Approval no. IACUC-2025122). As this study involved only animal subjects, no “consent to participate” was applicable.

#### 2.2.1 Mouse model of TLE

TLE model was established as previously described (16). On the day prior to modeling, lithium chloride (LiCl) was administered intraperitoneally to the mice at a dose of 180 mg/kg. After 17.5 h, methscopolamine bromide (MB) was injected intraperitoneally at 1.0 mg/kg to inhibit peripheral cholinergic effects and improve the survival rate of the mice. Thirty minutes later, pilocarpine (Pilo) was administered intraperitoneally at 300 mg/kg to induce TLE (17). We assessed the seizure severity in mice using the Racine scale, categorizing those with Grade IV or higher seizures or inducing status epilepticus (SE). It is considered as successful modeling (18). During subsequent experiments, mice with significantly deviant results or those that died were excluded from the analysis.

#### 2.2.2 Behavioral test

*Open field test (OFT)*, prior to the experiment, mice were placed in the testing environment for 12 h of habituation to adapt to the ambient light, temperature and other environmental conditions of the test room. The behavioral apparatus consisted of a white plastic open-field arena with dimensions of 50 × 50 × 40 cm. Using the Smart 3.0 animal behavior video analysis system, a central area of 15 cm × 15 cm was defined in the middle of the arena, and the remaining space was designated as the peripheral area. Each test lasted for 10 min. The number of entries into the central zone, residence time in the central area, and total moving distance were automatically recorded. After each trial, the bottom and inner walls of the arena were thoroughly wiped with 75% ethanol to eliminate residual odor interference between individual mice.

*Y-maze*, stage 1: The passages of all three arms of the Y-maze were fully opened without occlusion. Mice were gently placed in the central area and allowed to freely explore the entire maze for 10 min. Animal activities were continuously recorded by a video camera throughout the period. Stage 2 (training phase): One arm of the Y-maze was randomly blocked, leaving the other two arms accessible. Each mouse was placed in the maze center facing an open arm and permitted to explore freely for 5 min. The frequency of arm entries and residence time within open arms were recorded via video monitoring. Stage 3 (testing phase): All three arms were reopened to restore the complete structure of the Y-maze. The mouse was returned to the central area facing one familiar arm and allowed to explore for another 5 min. Exploratory behaviors were video-recorded, and the duration spent in the newly opened arm was statistically analyzed.

#### 2.2.3 Immunofluorescence

Immunofluorescence staining of the cellular proto-oncogene Fos (c-Fos) (rat antibody, 1:500, Synaptic Systems, Cat: 226017, RRID: AB_2864765) was performed to observe brain regions activated following acute TLE seizures.

#### 2.2.4 Mouse craniotomy

Mice were anesthetized with isoflurane (5% for induction, 2% for maintenance) and fixed on a stereotaxic instrument. First, the hair on the head was shaved, and the area was disinfected. A sagittal incision was made along the midline of the skull to expose the cranial surface, with the scalp, underlying tissue, and periosteum removed. A cranial window was drilled using an electric drill, spanning from 2 mm anterior to the anterior fontanelle to 10 mm posterior to it, with a width of 15 mm. Tweezers were then used to remove the skull segment while preserving the integrity of the dura mater. Care was taken during skull removal to avoid excessive bleeding, which could interfere with imaging results. Glue was then applied to fix a customized headband to the mouse’s head, ensuring it was secure and less likely to detach. The mice were allowed a 7-day postoperative recovery period. Throughout the surgical procedure, the body temperature of the mice was maintained at a constant 38 °C using a heating pad.

#### 2.2.5 Electrode implantation in the mouse cortex

Mice were anesthetized with isoflurane (5% for induction and 2% for maintenance) and securely fixed on a stereotaxic apparatus. The hair on the cranial vertex was removed, and the scalp skin was disinfected with iodophor, followed by a midline incision to expose the skull surface. Three small holes were drilled in the skull, and 1.2 mm-diameter screws connected with electrodes were implanted. Two screws were positioned over the cranial bone covering the cerebral cortex as recording electrodes, and the third was placed in the cerebellar region serving as the reference and fixation electrode. During surgery, erythromycin ophthalmic ointment was topically applied to the eyes to prevent corneal dryness and injury. After the operation, mice were transferred to a warming platform at 37 °C for rewarming. Upon full recovery from anesthesia, the animals were housed in individually ventilated cages. Subsequent experiments were initiated only after complete postoperative recovery.

### 2.3 The fUS experimental protocol

#### 2.3.1 Detection of CBV in mice

Typical experimental protocols included acquiring awake-state data from mice. From the environmental adaptation phase to the formal test, each mouse was recorded for 1 h at the same time daily to minimize stress responses during formal testing and maintain a relatively stable state during baseline recording. CBV reflects regional blood volume in the brain. In this experiment, resting-state CBV measurements obtained before the administration of normal saline or Pilo were averaged to establish the CBV baseline. CBV changes were measured over a total of 3,600 s: the first 1730 s served as the baseline period, 30 s for intraperitoneal drug injection, and the final 1800 s as the post-injection period. CBV values were directly exported from the software, and relative CBV (rCBV) was calculated as (CBV − CBV baseline)/CBV baseline.

#### 2.3.2 Resting-state functional connectivity detection

Resting-state FC data acquired from mice during the same period were segmented using the integrated Allen Mouse Brain Atlas in dedicated software (IcoStudio). This software enables accurate automatic registration and recognition of brain regions, facilitating the extraction of whole-brain CBV signals. To quantify connectivity patterns, Pearson correlation coefficients between spatially averaged, preprocessed CBV signals across all regions of interest (ROIs) were calculated (19). Blood signals were low pass filtered at 0.1 Hz; subsequently, Pearson correlation coefficients were used to generate a 12 × 12 correlation matrix. Mice received an intraperitoneal injection of dexmedetomidine (DEX; 0.05 mg/mL) to induce sedation with minimal spontaneous activity (20) prior to measurements. Correlation matrices for each trial were calculated over two time periods: a 1-h baseline recording, followed by an intraperitoneal Pilo injection and an additional one hour of recording. Then generate individual specific correlation matrices, and finally average the matrices of all mice.

A self-control design was adopted, where each mouse first received an intraperitoneal injection of normal saline (control condition), followed by an intraperitoneal injection of Pilo (TLE condition). The interval between the two experimental sessions was at least 24 h. Meanwhile, video monitoring was used to detect epileptic seizures in mice.

#### 2.3.3 Selection of ROIs

Brain regions selected for CBV recording were nuclei associated with epileptic seizures, as identified by c-Fos staining and reported in previous literature. These included the hippocampal CA1 (CA1) and CA3 (CA3) fields, dentate gyrus (DG) (21), anterior group of the dorsal thalamus (ATN) (22), intermediodorsal nucleus of the thalamus (IMD), reticular nucleus of the thalamus (RT) (23), posterior parietal association areas (PTLp) (24), primary motor area (MOp) (25), primary somatosensory area (SSp) (26), retrosplenial area (RSP) (27), secondary motor area (MOs) (28), and striatum (STR) (29). The names and abbreviations of these nuclei were derived from the Allen Brain Atlas (30).

### 2.4 Statistical analysis

To clarify the data analysis approach, the following procedures were adopted: Averages from all animals were calculated to determine the overall mean deviation for each experimental condition, and statistical analyses were performed on these averaged values. Paired *t*-tests were used to compare rCBV changes in the 12 ROIs during acute TLE seizures, with graphs generated using R software. Subsequently, paired-sample *t*-tests were used to statistically analyze the percentage of pixels showing positive and negative changes in mean rCBV. Pearson correlation coefficients between the 12 ROIs were calculated to assess pairwise nuclei correlations, with statistical analysis performed via *t*-tests. The following significance annotations were used: not significant (ns) for *p* > 0.05, *p < 0.05, **p < 0.01, ****p < 0.0001.

Whole-brain c-Fos immunofluorescence staining was performed on the mice to quantify the degree of neuronal activation in different brain regions. Image J software was used for counting c-Fos-positive neurons in the two groups of mice, and the counting was conducted in a blinded manner. For each mouse, consecutive non-overlapping sections of the target brain regions were selected, and 3–5 random fields of view were counted per section. Data were expressed as the mean number of cells per mm^2^ of brain region ± standard error of the mean (mean ± SEM). The counting criteria were defined as follows: neurons with clearly visible DAPI-labeled nuclei, and c-Fos immunofluorescence signal intensity significantly higher than the background, showing distinct punctate or patchy distribution.

EEG recordings were performed simultaneously during the establishment of the mouse TLE model using a biomedical signal acquisition and processing system (BL-4221). The low-pass filter was set at 100 Hz and the high-pass filter at 0.1 Hz. All signals were sampled at 2000 Hz with 16-bit analog-to-digital (AD) conversion. Spectral power was calculated separately for the following frequency bands: *δ* (0.5–3 Hz), *θ* (4–7 Hz), *α* (8–13 Hz), *β* (14–30 Hz), and *γ* (30–100 Hz).

## 3 Results

### 3.1 Establishment of a mouse model of acute seizure state of TLE

**Figure 1:**
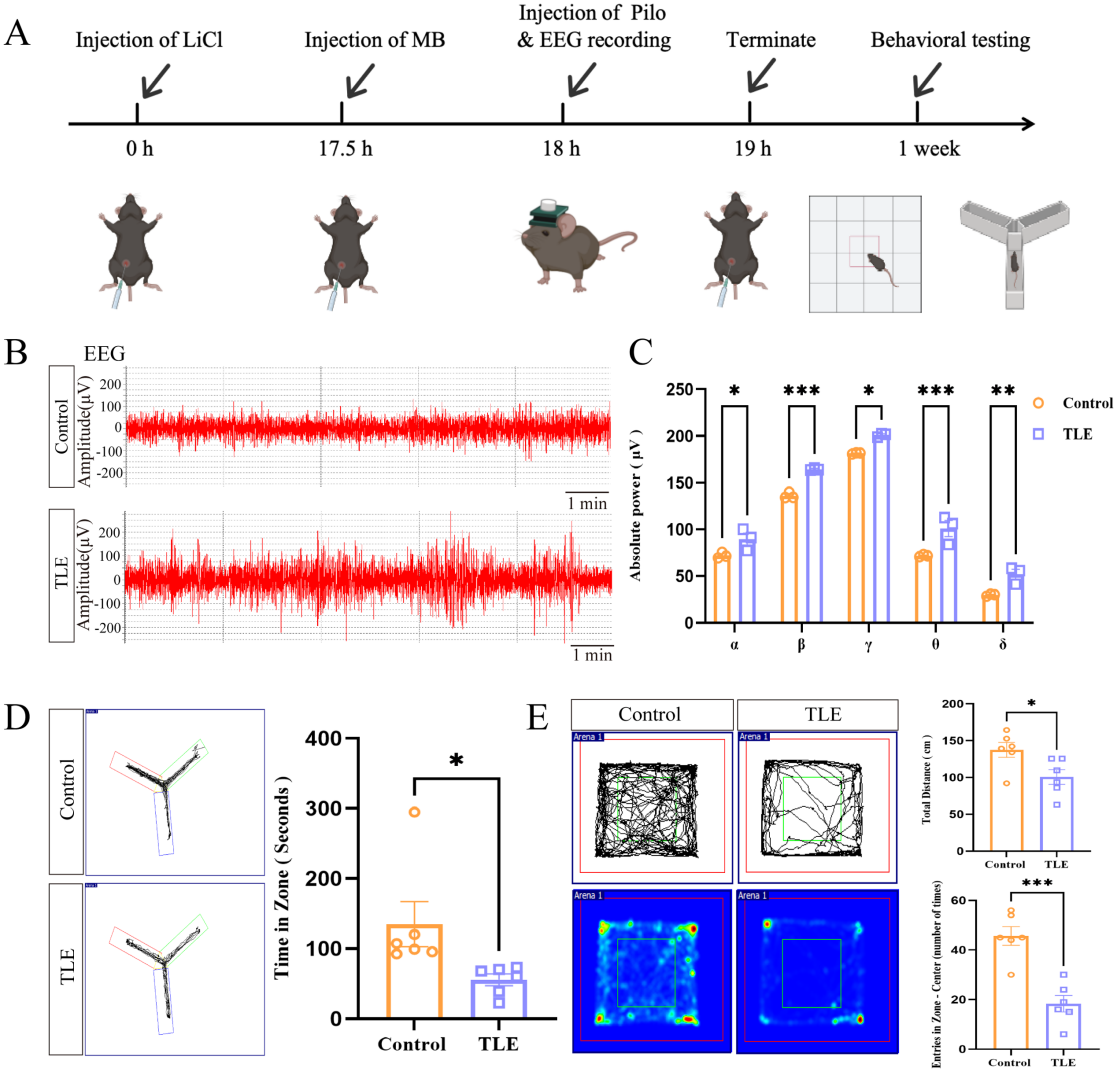
Establishment and validation of the mouse temporal lobe epilepsy (TLE) model. **(A)** Schematic illustration of the overall experimental protocol used for TLE model preparation and subsequent assessments. **(B)** Representative electroencephalography (EEG) traces recorded from mice in the two experimental groups. **(C)** Statistical comparison of distinct EEG waveform characteristics between groups, analyzed by Two-way ANOVA; n = 3 per group. **(D, E)** Behavioral assessments of locomotor and cognitive function using the open field test (D) and Y-maze test **(E)**, with group comparisons performed by t-test; n = 6 per group. All mouse illustrations and behavioral task schematics were created using BioRender. Data are presented as mean ± standard error of the mean (SEM); *P < 0.05, **P < 0.01, ***P < 0.001.

A LiCl-Pilo-induced TLE mouse model was established, with all drugs administered via intraperitoneal injection. Acute seizure state of TLE in mice were observed, and their EEG were recorded. Only mice exhibiting TLE seizures of stage IV or higher were included in subsequent experiments. One week later, behavioral tests [open field test (OFT) and Y-maze test] were conducted. The modeling protocol is depicted in Figure 1A. Electroencephalographic recordings (Figure 1B) revealed distinct spikes in the TLE group during acute seizures, which were absent in the control group. Additionally, Figure 1C shows that the frequency of various waveforms in the TLE group increased to varying degrees following seizures. Behavioral assessments further supported these findings: in the Y-maze test (Figure 1D), where the red box indicates the newly opened arm (arm A), TLE mice exhibited a significantly reduced latency to enter this arm. In the open field test (Figure 1E) the TLE group displayed a shorter total movement distance and fewer entries into the central area compared to controls. Collectively, these results confirm the successful establishment of the mouse TLE model and the demonstrate the presence of TLE-induced cognitive impairment.

### 3.2 Changes in CBV during acute TLE seizures in mice

**Figure 2:**
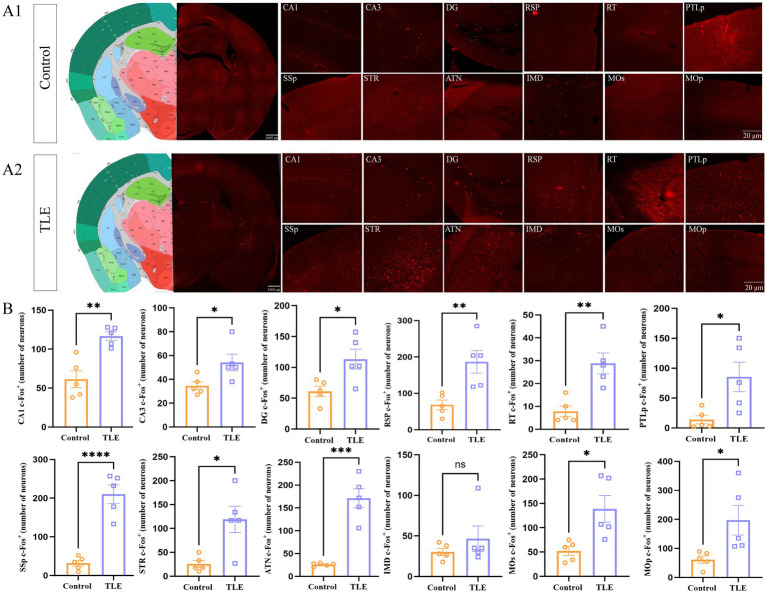
Comparative analysis of c-Fos immunofluorescence staining between control and temporal lobe epilepsy (TLE) mouse groups. **(A1, A2)** Representative c-Fos immunofluorescent images showing c-Fos+ cells in 12 distinct brain regions from control and TLE mice. **(B)** Quantitative comparison of c-Fos immunoreactivity between the two groups, with representative immunofluorescent images and corresponding statistical analysis. Data were analyzed using a t-test; n = 5 per group; data are presented as mean ± SEM; *P < 0.05, **P < 0.01, ***P < 0.001.

**Figure 3:**
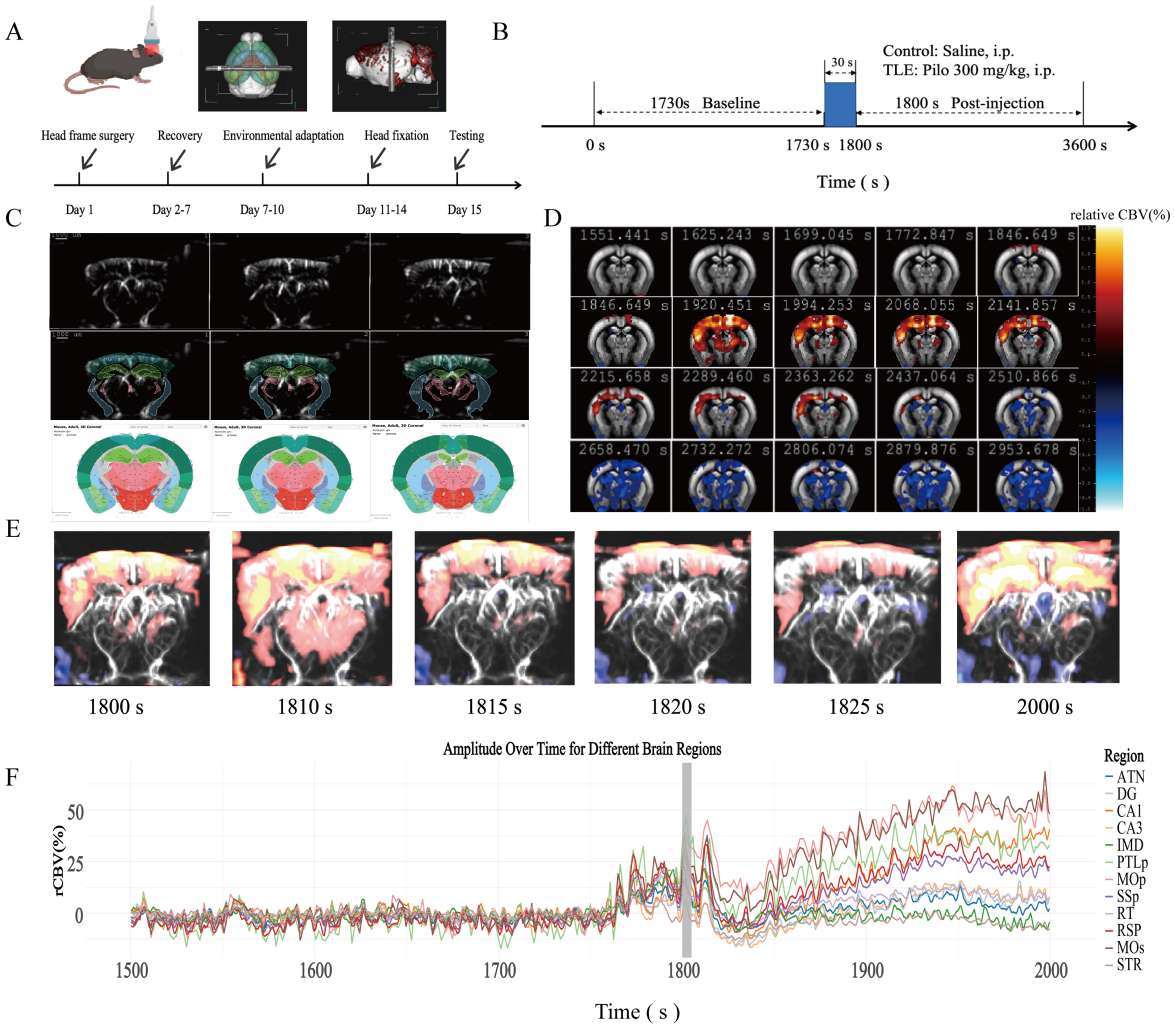
Regional relative cerebral blood volume (rCBV) changes in a mouse model of temporal lobe epilepsy (TLE). **(A)** Schematic of the experimental setup for functional brain imaging and the corresponding brain regions examined. **(B)** Timeline of the functional brain imaging experimental protocol. **(C)** Representative brain slice used for blood flow detection, adapted from the Allen Mouse Brain Atlas. **(D)** Functional ultrasound Doppler images illustrating whole-brain rCBV alterations during acute TLE seizures; red indicates increased blood flow, whereas blue indicates decreased blood flow. **(E)** Temporal dynamic changes in whole-brain rCBV, showing peak blood flow elevation during acute seizures at 1800–2000 s. **(F)** Quantitative rCBV changes across 12 selected brain regions; the line graph was generated using R software.

First, whole-brain c-Fos staining was performed during acute seizure state of TLE in mice, revealing activation of several brain regions, including CA1, CA3, DG, RSP, RT, PTLp, SSp, STR, ATN, MOs, and MOp (Figures 2A,B). Subsequently, fUS technology was used to investigate acute changes in CBV during acute seizure state of TLE in mice. Three-dimensional (3D) images of mouse heads showed CBV measurement levels from multiple angles. On day 1, mice underwent craniotomy and head frame implantation. After a 7-day recovery period, mice were placed on a custom-made rack for environmental adaptation from days 7 to 10. From days 11 to 14, the ultrasound probe position was adjusted and coupled to the mice’s heads using ultrasound gel, with formal measurements conducted on day 15 (Figure 3A). The control group received intraperitoneal injection of saline, while the TLE group received Pilo. CBV maps at three detection levels were matched with the Allen Brain Atlas (Figures 3B,C). Figure 3D illustrates changes in rCBV during acute seizure state of TLE in mice: rCBV remained unchanged at baseline; after TLE onset, rCBV increased significantly before decreasing, with these changes predominantly localized to the cortex, hippocampus, and thalamus. The peak rCBV occurred between 1,800 and 2,000 s. After 2,500 s, the overall rCBV decreased significantly, indicating hypoperfusion. Figures 3E,F further illustrate whole-brain rCBV changes within the 1,800–2,000 s window.

**Figure 4:**
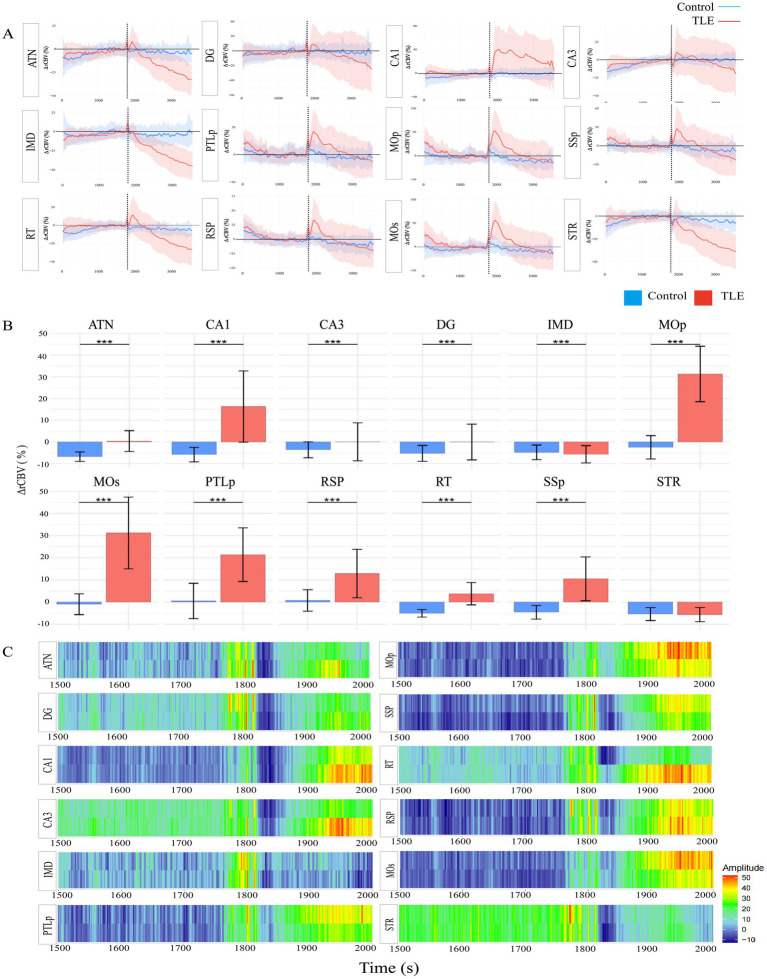
Temporal differences in relative cerebral blood volume (rCBV) changes across brain regions. **(A)** Temporal trajectories of rCBV in 12 seizure-related brain regions; blue line represents the control group and red line represents the TLE group. The plot was generated using R software. **(B)** Quantitative comparison of rCBV in the 12 brain regions during the peak seizure period (1800–2000 s). Group differences were analyzed by t-test. **(C)** Heatmap showing temporal differences in rCBV across brain regions during 1800–2000 s, with 1500–1730 s as the baseline period. Data are presented as mean ± SEM; n = 7 per group; *P < 0.05, **P < 0.01, ***P < 0.001.

rCBV values for the 12 ROIs were calculated and plotted using R software, with the blue line representing the saline-injected control group and the red line representing the TLE group. Results showed that acute seizure state of TLE, rCBV in all nuclei except IMD and STR exhibited a brief significant increase followed by a decreasing trend. Most brain regions showed a blood flow peak at approximately 2,000 s, with CA1 displaying robust and sustained blood flow (Figure 4A). Of note, preliminary experimental results revealed that the differences in rCBV values among individual mice were negligible. Accordingly, we averaged the rCBV data from seven mice to generate the results presented in Figure 4A. The raw individual rCBV data of each mouse have been provided in the Supplementary materials. Subsequent statistical quantification of rCBV changes in each brain region during 1,800–2,000 s revealed the following order of blood flow increase: MOp, MOs, PTLp, CA1, RSP, SSp, RT, CA3, ATN, DG, IMD, STR (Figure 4B). Additionally, temporal changes in rCBV varied slightly across brain regions. The heatmap showed that RT blood volume changes occurred within 1,800–2,000 s, earlier than in other regions, followed by changes in the cortex and hippocampus (Figure 4C). Notably, rCBV in IMD and STR did not change significantly and even showed a sustained decrease.

### 3.3 Positive and negative subgroup responses during acute seizure state of TLE

**Figure 5:**
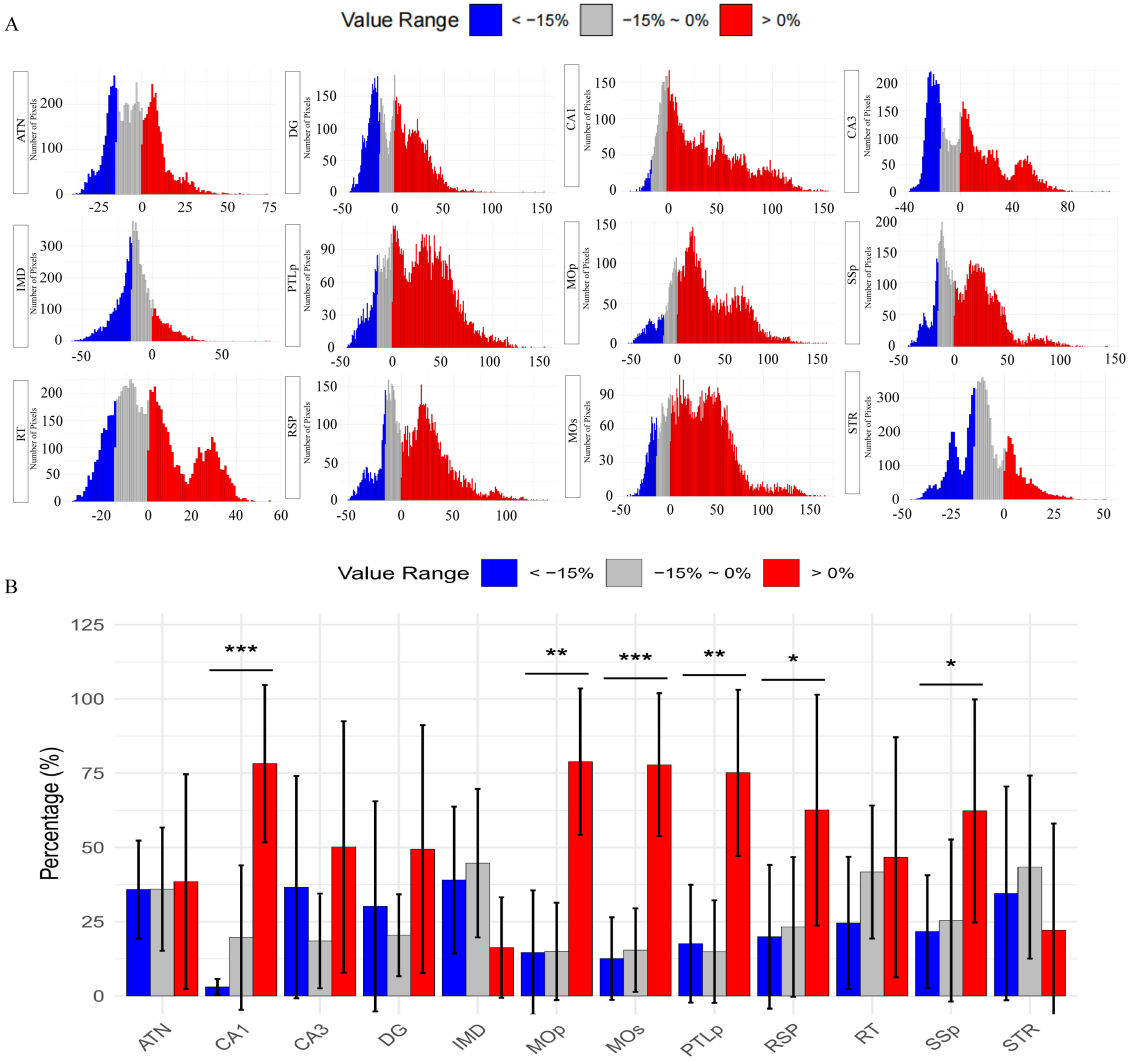
Pixelbased analysis of pilocarpineinduced relative cerebral blood volume (rCBV) changes in the mouse temporal lobe epilepsy (TLE) model, identifying positive and negative response subgroups. **(A)** Pixel plot illustrating mean rCBV changes across 12 brain regions during acute TLE seizures (1800–2500 s) relative to baseline. **(B)** Statistical comparison of the proportion of pixels displaying distinct magnitudes of mean rCBV change. Data were analyzed using a pairedsample ttest; n = 7 per group; data are presented as mean ± SEM; *P < 0.05, **P < 0.01, ***P < 0.001.

In the preceding results, each brain region was treated as an integrated unit, where rCBV at each time point was defined as a single pixel. Pixel values for each nucleus were then averaged across all experimental mice. As shown in the heatmap, during acute seizure state of TLE in mice, each brain region exhibited both positive and negative blood volume responses. Subsequently, rCBV changes of all pixels within each brain region were calculated for the period of between 1,800 and 2,500 s, and the data were summarized. During the initial 1,730 s of baseline recording, global rCBV fluctuations were primarily confined to the range of −15% to 0, serving as a reference for rCBV variability during the awake state. Based on pixel-level rCBV changes, responses were categorized into three groups: negative responses (< −15%, blue), no significant responses (−15% to 0, gray), and positive responses (>0, red). The total number of pixels in each response category was counted and compared (Figure 5A). Subsequently, statistical analysis was performed (Figure 5B). The results revealed that in hippocampal regions (CA1, CA3, DG), motor cortex (MOp, MOs), RSP, PTLp, SSp, and RT, the number of positive responses significantly exceeded that of negative responses. In the ATN, the numbers of positive and negative responses were comparable. In the IMD and STR, however, the number of negative responses was significantly higher than that of positive responses.

### 3.4 Changes in brain functional network connectivity associated with acute seizure state of TLE

**Figure 6:**
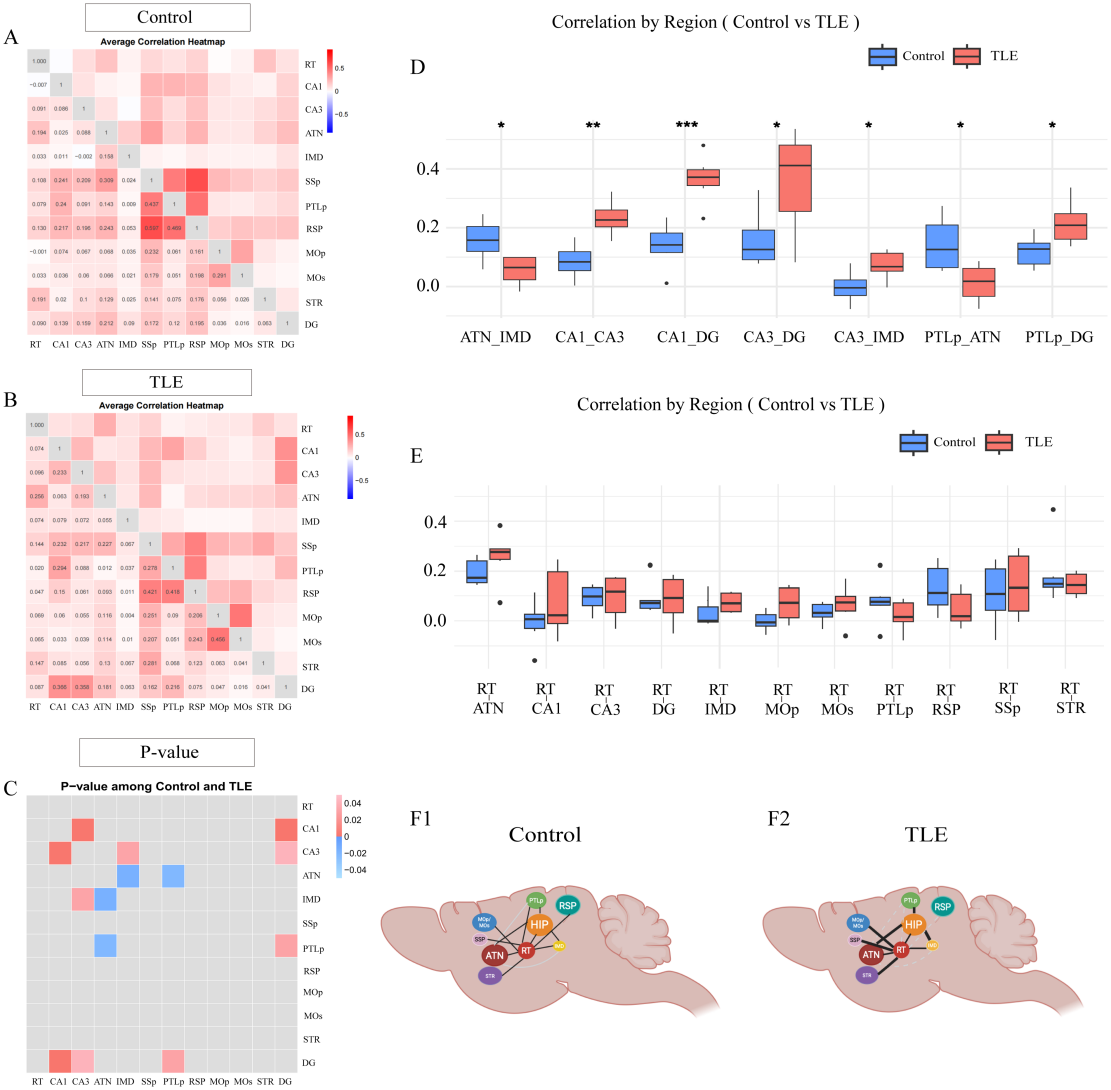
Acute TLE seizures disrupt cerebral hemodynamic correlation and functional connectivity (FC) in mice, with prominent alterations in the cortex, hippocampus, and thalamus. **(A–C)** FC changes among 12 brain regions during acute TLE seizures. **(D)** Correlation changes between the cortex, hippocampus, thalamus, and seizurerelated nuclei. **(E)** Differential FC between the reticular thalamus (RT) and other selected nuclei. **(F)** Wholebrain FC network diagrams; line diameter indicates global connectivity differences, edge width and color intensity represent FC changes between brain regions, and gray dashed lines denote reduced FC. Data were analyzed by ttest; n = 6 per group; data are presented as mean ± SEM; *P < 0.05, **P < 0.01, ***P < 0.001. This panel was created using BioRender.

Cerebral hemodynamic correlations for each region of interest were calculated in both groups during the baseline and post-injection periods. The average Pearson correlation coefficient matrix of rCBV changes in the 12 epilepsy-related brain regions, along with intergroup differences in *p*-values, was analyzed (Figures 6A–C). The TLE group exhibited enhanced FC involving multiple pairs of brain regions. Specifically, the overall connectivity of RT shows an increasing trend (Figure 6E). There was a positive correlation among the various subregions of the hippocampus (CA1-CA3, CA1-DG, CA3-DG). Significant enhancement of this positive correlation was also observed between the cortex and hippocampal regions (PTLp-DG), as well as between hippocampal regions and the thalamus (CA3-IMD). Conversely, significant negative correlations were detected between thalamic nuclei (ATN-IMD) and between the cortex and thalamus (PTLp-ATN) (Figure 6D). Figure 6F1 depicts the FC map of the control group, while Figure 6F2 represents that of the TLE group.

## 4 Discussion

In this study, we established an experimental protocol using fUS to record CBV in mice and assess FC, thereby revealing the correlation between blood flow response patterns and hemodynamics during acute seizure state of TLE. Our findings indicate that most brain regions—including the cortex, hippocampus, and thalamus—exhibit changes in rCBV. Specifically, among the 12 epilepsy-related brain regions, all except the IMD and STR showed an initial increase followed by a decrease in rCBV. Additionally, the heatmap demonstrates that rCBV changes in the RT occur earlier than in other nuclei. Moreover, the brain^’^s FC undergoes distinct changes, which may be associated with the remodeling of brain FC during this period.

### 4.1 The measurements of rCBV and FC involved two independent experimental protocols

We fully recognize the well-documented effects of anesthetics and sedatives on neural activity and neurovascular coupling. To address this critical issue, low-dose dexmedetomidine (a highly selective α2-adrenergic agonist) was used for sedation in the present study (31). This administration regimen has been fully validated in previous studies, as it can steadily maintain physiological functional connectivity (FC) and stimulus-evoked neural responses in the mouse brain, preserve the integrity of neurovascular coupling, and minimize motion artifacts and stress responses. Considering that the detection of FC via functional ultrasound imaging (fUS) relies on capturing subtle microvascular dynamic changes, a relatively stable physiological state of animals is strictly required. Accumulated evidence has demonstrated that dexmedetomidine at clinically relevant sedative doses does not suppress global FC or abrogate task-dependent network modulation. Therefore, mild sedation was applied to mice during FC measurements.

In contrast, to recapitulate the physiological characteristics of acute epileptic seizures, relative rCBV detection and analysis were performed in awake mice. Notably, these two assessments originate from independent experimental designs, and we have provided detailed explanations for this methodological distinction in the Discussion section. Furthermore, our future work will focus on establishing optimized approaches for awake FC recording to overcome the current technical limitations.

### 4.2 Biphasic hemodynamic responses during acute seizure state of TLE

Compared with the previous use of c-Fos immunofluorescence staining to identify seizure-related brain regions, the present study found that rCBV in the IMD and STR continued to decrease. In contrast, rCBV in the ATN, DG, CA1, CA3, PTLp, MOp, MOs, RSP, RT, and SSp exhibited a biphasic pattern (initial increase followed by decrease), with variations in the amplitude and temporal dynamics of the initial increase. We divided the hemodynamic changes during acute seizure state of TLE into two distinct stages. The first stage corresponds to a surge in cerebral metabolic demand (32, 33). In response, cerebral blood vessels undergo compensatory dilation, resulting in increased CBV—a finding consistent with previous research (34). During generalized seizures, the entire brain enters a high-perfusion state. The second stage occurs when brain tissue transitions to an inhibitory state after seizure termination, causing a sudden reduction in metabolic demand (35). Persistent electrical activity leads to ATP depletion, impairing cerebrovascular regulatory capacity and inducing decompensation of the neurovascular coupling mechanism. Consequently, local blood flow fails to meet metabolic requirements (36), resulting in sustained CBV decrease and ultimately cerebral ischemic injury. Taken together, neuronal damage and blood flow changes interact reciprocally, eventually causing the loss of compensatory mechanisms, further neuronal damage, and continuous CBV decrease—forming a vicious cycle.

### 4.3 Changes in brain functional networks during acute seizure state of TLE

We analyzed correlations among the 12 brain regions previously evaluated for seizure-associated CBV changes. Previous studies have shown that activation or inhibition of the RT via electrical stimulation or optogenetics significantly suppressed or exacerbated epileptic seizures, suggesting that the RT may serve as a physiological target for clinical epilepsy treatment (23, 37). Our experimental results indicate that the correlation between the RT and the cortex, hippocampus, and thalamus was enhanced to varying degrees. Positive correlations were observed between the cortex and hippocampus, and between the hippocampus and thalamus. Significant negative correlations were detected between thalamic nuclei and between cortical-thalamic pairs. These findings reflect functional brain network connectivity may exhibit adaptive dynamic remodeling.

### 4.4 Validation of correlations between current study results and previous research

Cerebrovascular disease is the most common cause of epilepsy (2). In addition, preserving vascular integrity represents a supplementary strategy to traditional antiepileptic drugs (38). During seizures, blood–brain barrier (BBB) integrity is impaired, and metabolic clearance is compromised (5, 33, 39), ultimately inducing hemodynamic dysfunction and CBV changes. Previous studies using brain functional imaging have reported a significant increase in CBV during seizures (4, 40), while another study noted reduced CBV during acute seizures in TLE patients (3)—two seemingly contradictory findings. Integrating our results with these previous observations indicates that rCBV first increased and then decreased. The current study specifically characterized CBV changes in epilepsy-related nuclei in awake mice during TLE and performed statistical analysis, identifying the temporal dynamics of CBV changes in these nuclei and the sequence of CBV change amplitudes—key distinctions from previous studies. Second, we characterized the previously underreported IMD; our results showed that IMD-CBV decreased continuously during TLE seizures. Previous literature has reported relatively little on the IMD. This suggests that during the acute stage of temporal lobe epilepsy seizures, the IMD represents a key thalamic subregion with prominent hemodynamic abnormalities. The IMD may specifically participate in the circuit regulation underlying acute epileptic processes. Nevertheless, these findings are limited to phenomenological observations at the cerebral perfusion level, and direct evidence regarding its precise regulatory functions and molecular mechanisms remains lacking. Additionally, the CBV of STR decreased during acute TLE seizures. In the previous c-Fos results, the STR was significantly activated. We speculate this is because the STR contains abundant GABAergic neurons (41), leading to reduced blood flow during acute seizure state of TLE.

Previous fMRI studies have shown that the strength of FC between the seizure focus and the treatment site of ANT-DBS correlates with seizure outcomes: stronger connectivity is associated with better clinical efficacy (42). Additionally, studies have demonstrated that functional changes in brain regions distant from the resection site following TLE surgery result from structural disconnections between these regions and the resected epileptic lesions (43). Our study characterized changes in brain functional networks during acute seizure state of TLE onset, elucidating the relationship between epilepsy and brain FC changes from multiple perspectives.

### 4.5 Limitations and future considerations

No separate interventional control group treated with saline only was established in this study. The adoption of a self-control design can minimize the interference of individual animal differences on detection results, as inherent physiological variations widely exist among mice. If an independent control group were introduced, such intrinsic inter-individual differences would directly compromise the accuracy of cerebral blood flow detection and cause biases in intergroup comparisons. Additionally, several previous studies have adopted a before-and-after self-control design (20), in which comparisons of the same animal at different time points effectively eliminate the confounding effects of individual heterogeneity on experimental outcomes. In terms of experimental statistical design, an independent animal control group treated solely with saline was not established in the present study. Although individual differences were controlled via a self-control design, and time-dependent effects and repeated measurement biases were excluded based on previous relevant studies, the potential impacts of long-term physiological fluctuations in individuals’ cerebral blood flow measurements could not be completely eliminated. Furthermore, the self-control design only reflects changes within the same animal under different treatments and fails to directly evaluate the influence of inter-individual population differences on experimental outcomes. In future research, we will supplement an independent control group and adopt a combined design of self-control and inter-group control to further validate the reliability of the conclusions drawn in this study.

Static FC was only assessed in anesthetized mice; thus, we cannot rule out the possibility that anesthetics may suppress neuronal activity, leading to differences compared to awake mice. On the other hand, we used a single Pilo injection dose (300 mg/kg) based on literature reports and did not assess CBV changes across different doses. Additionally, we only observed changes in CBV and brain FC during acute seizure state of TLE (30 min to 1 h) and did not evaluate CBV changes during chronic TLE, which may limit the generalizability of our findings.

## 5 Conclusion

In conclusion, this study utilized focused fUS technology to characterize the patterns of CBV changes and hemodynamic correlations during acute seizure state of TLE. We observed a biphasic CBV pattern (initial increase followed by decrease) in most epilepsy-related brain regions. Differences were identified in the amplitude and temporal dynamics of blood volume changes across various brain regions. Additionally, pixel-based analysis revealed positive and negative CBV responses in distinct brain regions, suggesting that the effects exerted differ among neuron types. Furthermore, significant changes were observed in whole-brain static FC: correlations between the hippocampus and thalamus, as well as between the hippocampus and cortex, were enhanced. Meanwhile, correlations between the RT and both cortical and hippocampal regions were increased, whereas correlations between the thalamus and certain cortical nuclei were reduced. These findings indicate that acute epileptic seizures can be accompanied by adaptive changes in brain network connectivity patterns.

Finally, this study demonstrates that fUS is a highly valuable *in vivo* imaging tool capable of revealing correlations between cerebral hemodynamics and epilepsy with high resolution.

## Data Availability

The original contributions presented in the study are included in the article/Supplementary material, further inquiries can be directed to the corresponding authors.
