# Supplementary material for: Functional ultrasound assessment of cerebral blood flow and brain connectivity in a pilocarpine-induced acute epileptic seizures in mice
Source: Front Neurol. 2026 Jun 15;17:1739882. doi: 10.3389/fneur.2026.1739882 (PMC13310741; doi:10.3389/fneur.2026.1739882)
Supplement: Supplementary file 1 [file Supplementary_file_1.PDF]

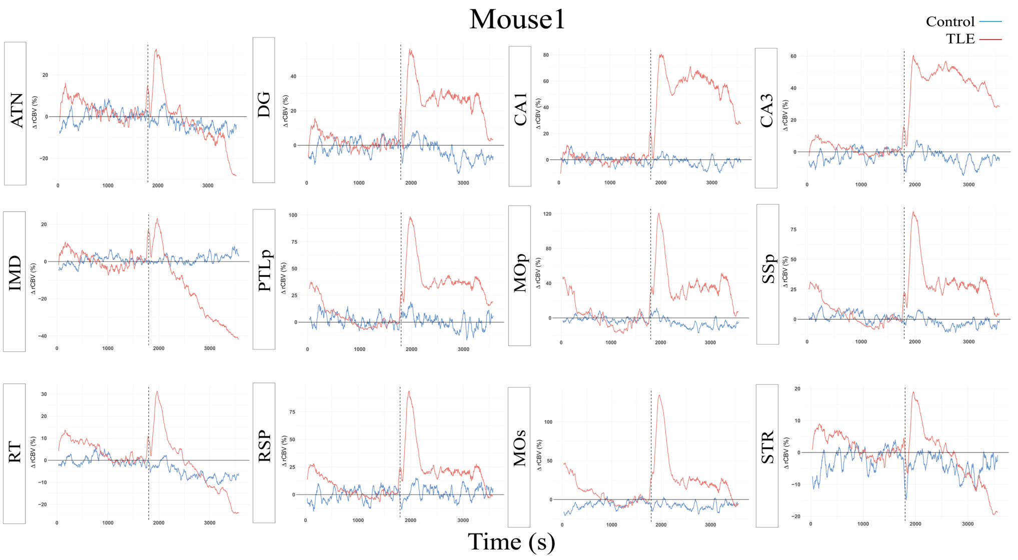
Mouse1
Control
TLE
ATN
DG
CA1
CA3
IMD
PTLp
MOp
SSp
RT
RSP
MOs
STR
Time (s)

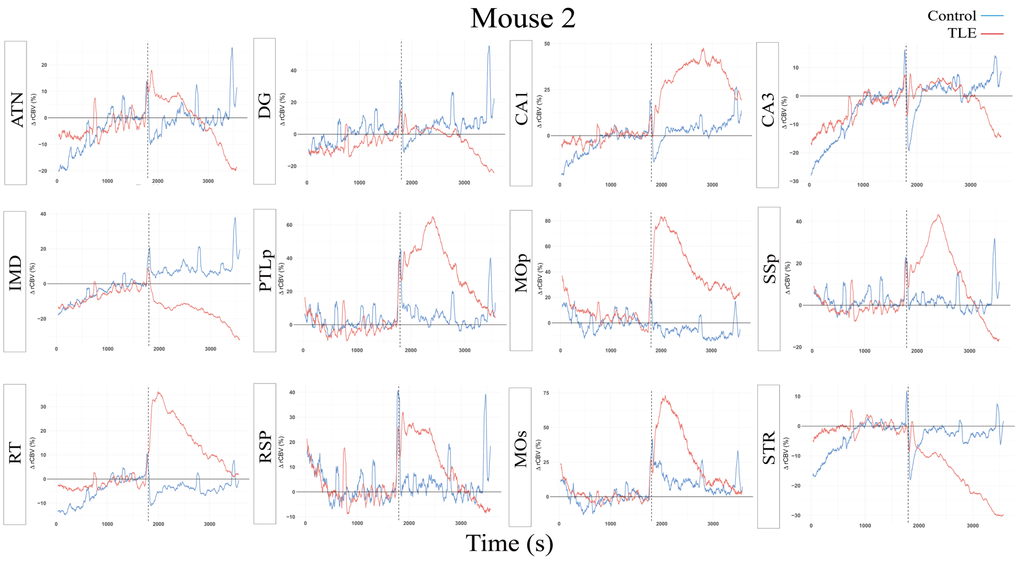
Mouse 2
Control
TLE
ATN
DG
CA1
CA3
IMD
PTLp
MOp
SSp
RT
RSP
MOs
STR
Time (s)

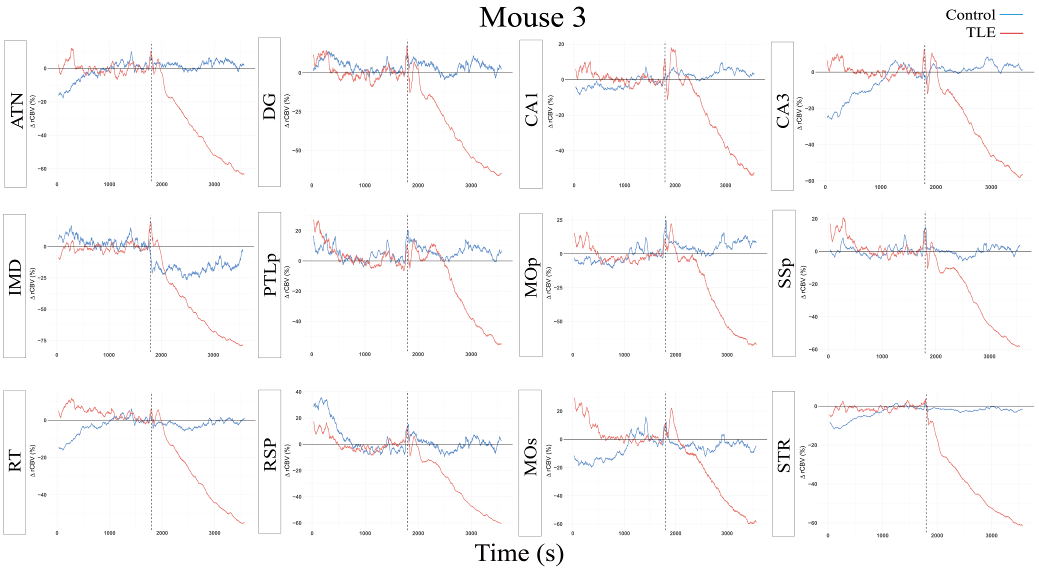
Mouse 3
Control
TLE
ATN
DG
CA1
CA3
IMD
PTLp
MOp
SSp
RT
RSP
MOs
STR
Time (s)

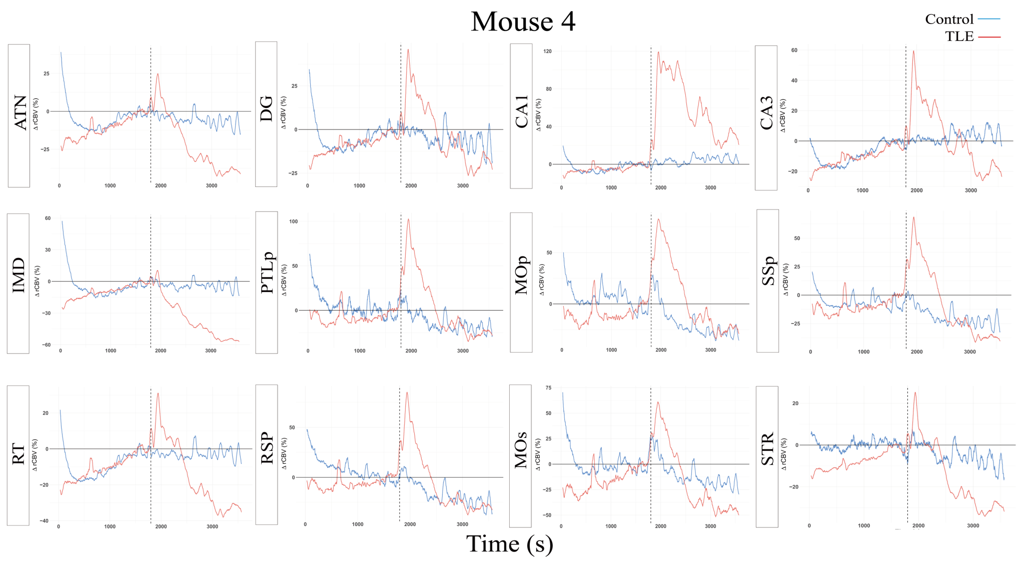
Mouse 4
Control
TLE
ATN
DG
CA1
CA3
IMD
PTLp
MOp
SSp
RT
RSP
MOs
STR
Δ rCBV (%)
Time (s)

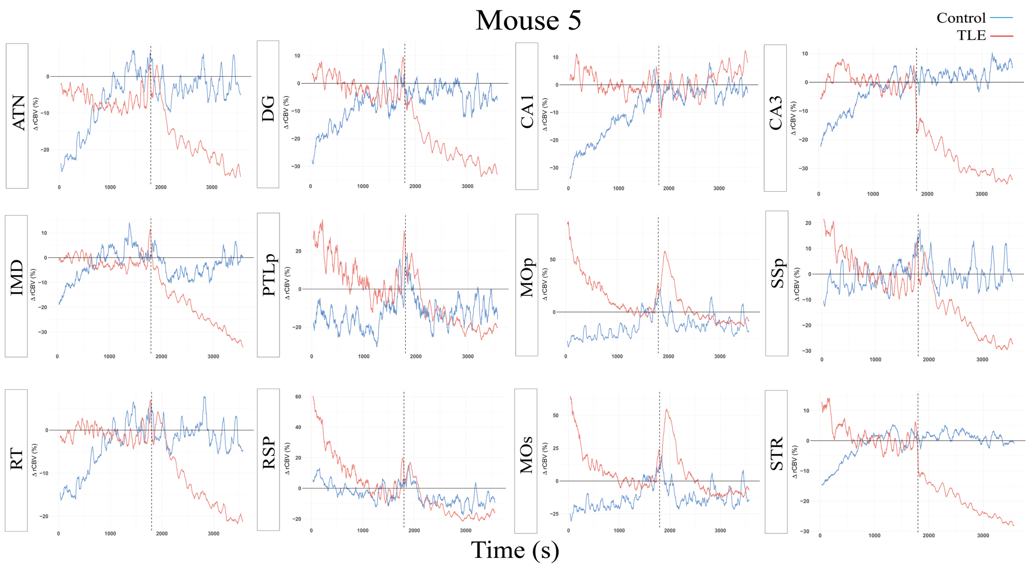
Mouse 5
Control
TLE
ATN
DG
CA1
CA3
IMD
PTLp
MOp
SSp
RT
RSP
MOs
STR
Δ rCBV (%)
Time (s)

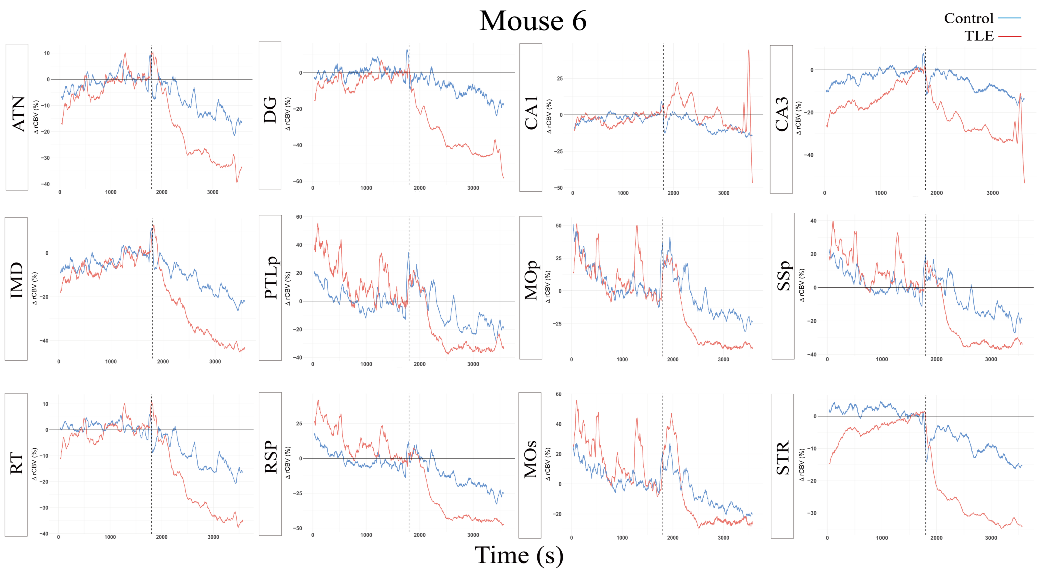
Mouse 6
Control
TLE
ATN
DG
CA1
CA3
IMD
PTLp
MOp
SSp
RT
RSP
MOs
STR
Δ rCBV (%)
Time (s)

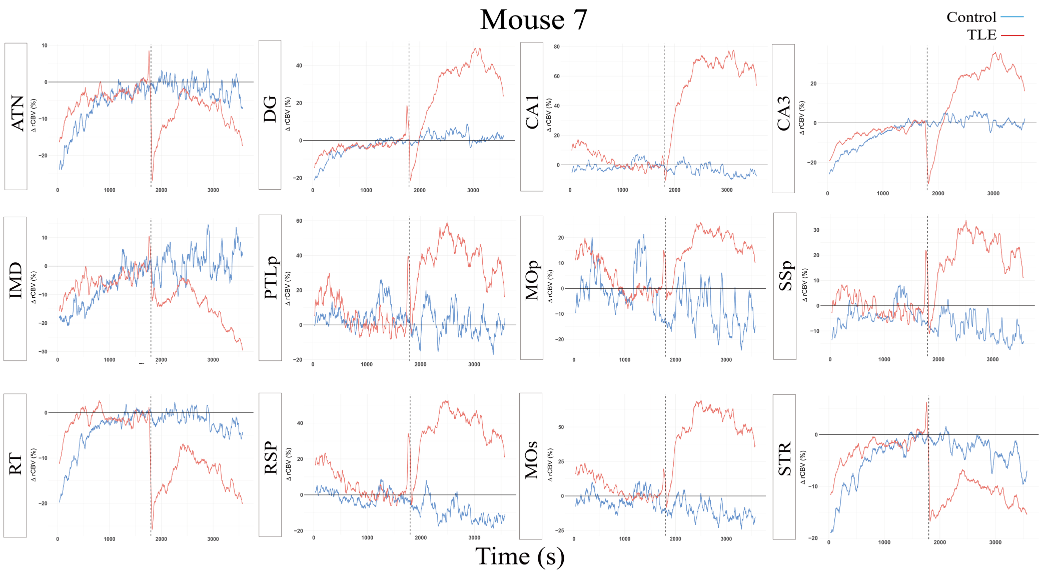

Supplementary Figure 1. Changes in relative cerebral blood volume (rCBV) across 12 brain regions in seven mice, blue line represents the control group and red line represents the TLE group. The plot was generated using R software, n = 7 per group, rCBV was calculated as (CBV − CBV baseline) / CBV baseline.
